# Utilizing the National Basketball Association’s COVID-19 restart “bubble” to uncover the impact of travel and circadian disruption on athletic performance

**DOI:** 10.1038/s41598-020-78901-2

**Published:** 2020-12-11

**Authors:** Andrew W. McHill, Evan D. Chinoy

**Affiliations:** 1 Oregon Institute of Occupational Health Sciences, Oregon Health and Science University, Portland, OR 97239 USA; 2 Leidos, Inc., San Diego, CA 92106 USA

**Keywords:** Circadian rhythms and sleep, Human behaviour

## Abstract

On March 11th, 2020, the National Basketball Association (NBA) paused its season after ~ 64 games due to the Coronavirus 2019 (COVID-19) outbreak, only to resume ~ 5 months later with the top 22 teams isolated together (known as the “bubble”) in Orlando, Florida to play eight games each as an end to the regular season. This restart, with no new travel by teams, provided a natural experiment whereby the impact of travel and home-court advantage could be systematically examined. We show here that in the pre-COVID-19 regular season, traveling across time zones reduces winning percentage, team shooting accuracy, and turnover percentage, whereas traveling in general reduces offensive rebounding and increases the number of points the opposing (home) team scores. Moreover, we demonstrate that competition in a scenario where no teams travel (restart bubble) reduces the typical effects of travel and home-court advantage on winning percentage, shooting accuracy, and rebounding. Thus, home-court advantage in professional basketball appears to be linked with the away team’s impaired shooting accuracy (i.e., movement precision) and rebounding, which may be separately influenced by either circadian disruption or the general effect of travel, as these differences manifest differently when teams travel within or across multiple time zones.

## Introduction

The internal circadian clock dictates the daily timing of changes in physiology and behavior, and has been found to impact even high-level activities such as athletic performance^1,2^. However, in modern society with the ability to easily travel across time zones, humans often initiate athletic activities at times when the internal clock may not be promoting optimal performance and thus could provide a benefit to the team that does not travel^3–5^. While colloquially referred to as “home-court advantage,” specific reasons for this advantage are often difficult to disentangle due to multiple variables pertaining to game-situations/travel occurring simultaneously (e.g., home-crowd noise, air-travel discomfort, time zone changes, etc.). Thus, natural experiments must be utilized to uncover the impact of travel on elite athletic performance. On March 11th, 2020, the National Basketball Association (NBA) paused its 2019–2020 season after ~ 64 games due to the novel Coronavirus 2019 (COVID-19) outbreak, only to resume ~ 5 months later with the top 22 teams isolated together in Orlando, Florida to play eight games each as an end to the regular season. By creating this “bubble,” the NBA generated a natural experiment whereby the impact of travel and time zone changes (which can cause circadian disruption) could be systematically teased apart.

In addition to the circadian advantages that a home-team may experience by not traveling, other factors such as sleep impairment by the away team could also contribute to the advantages of playing at home. In fact, many NBA players report that frequent travel and associated travel-related discomforts cause sleep loss and fatigue, negatively affecting their health, performance, recovery, and mood/mindset^6–8^. On a physiological level, sleep and wakefulness are either promoted or inhibited at different times of day by an interaction between a homeostatic build-up (i.e., sleep pressure) related to prior wake duration and the phase (i.e., timing) of the near 24-h circadian clock^9,10^. When traveling across time zones, the sudden shift in timing of one’s activity and exposure to the new environmental light–dark cycle causes a desynchrony in the central circadian clock located in the suprachiasmatic nucleus of the hypothalamus^11^, which alters the phase relationship between sleep and circadian timing. The circadian clock must then re-synchronize to the new time zone^12^, and the resulting jet-lag is a major factor that subsequently disturbs sleep^13^. Thus, the frequent travel within and between time zones required of players across the NBA season (6+ months) can both chronically and acutely disrupt sleep and circadian rhythms. However, these disruptions are likely to affect game playing performance and winning percentage to varying magnitudes at different points across the season, depending on the team’s recent stability (home games) or disruption (away games) in their schedule.

Utilizing the unique context of the 2019–2020 NBA season, we therefore aimed to examine how travel and time zone changes affect team regular season winning and athletic performance.

## Results

**Figure 1:**
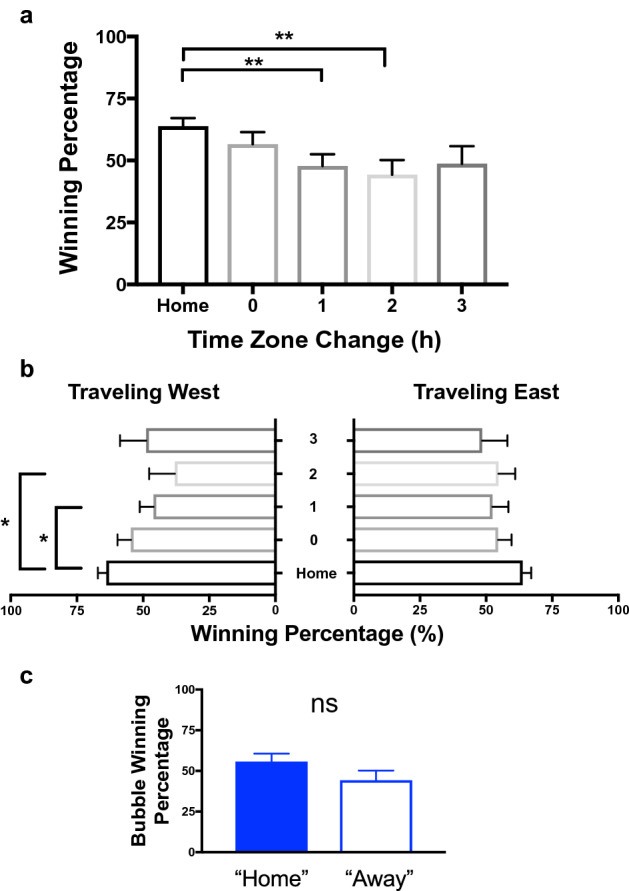
Team winning percentage when playing at home, away (by time zone change), and in the restart bubble with no travel. Brackets with asterisks denote significant differences at the end of each line; * denotes p < 0.029, **denotes p < 0.01. NS denotes not significant. Error bars are standard error of the mean.

Prior to the restart, teams won 63.8% of their home games, which was significantly higher than their 50.8% away game winning percentage (t(21) = 3.6, p < 0.01). Winning percentage (Fig. 1a) differed depending on time zone (F(4) = 2.74, p = 0.03), with planned comparisons revealing that compared with home games, winning percentage only differed for away games when traveling across one (t(21) = 2.98, p = 0.007) or two (t(21) = 3.18, p = 0.005) time zones, was a non-significant trend for lower winning percentage when traveling three time zones (t(11) = 2.25, p = 0.04), and was not different when traveling but staying within their home time zone (t(21) = 1.84, p = 0.08). There was no significant relationship between the overall number of time zones a team crossed when traveling pre-COVID-19 and away winning percentage (r(20) = 0.14, p = 0.53). Moreover, winning percentage differed depending on the direction of travel (Fig. 1b), with teams traveling westward losing significantly more than at home (F(4) = 2.87, p = 0.03), specifically at one (t(17) = 2.44, p = 0.026) and two (t(14) = 2.61, p = 0.02) time zones away, and eastward travel having no significant effect (F(4) = 1.59, p = 0.20). However, when teams played within the bubble—but still being denoted as either the home or away team, albeit with no travel or changing time zones and only virtual fans—there was not a significant difference in home vs away winning percentage (55.7% vs 44.3%; t(21) = 1.9, p = 0.07; Fig. 1c).

**Figure 2:**
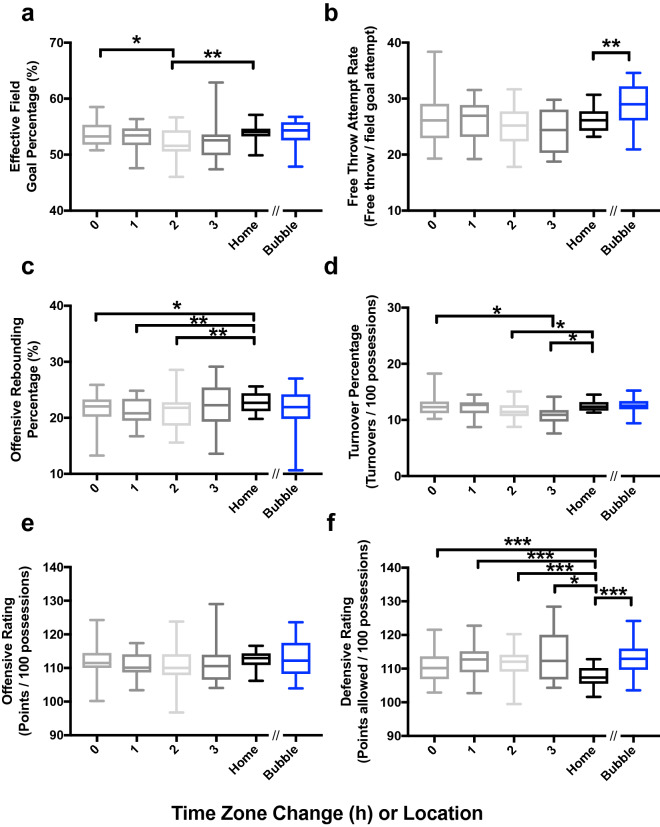
Advanced in-game metrics when playing at home and away (by time-zone change) in pre-COVID-19 games, and all games (combined home and away) in the restart bubble with no travel. The bubble games were only compared with the pre-COVID-19 home games. Brackets with asterisks denote significant differences at the end of each line; *denotes p < 0.029, **denotes p < 0.01, ***denotes p < 0.001. Error bars are standard error of the mean.

To uncover the specific impact of travel and time zone changes on in-game athletic performance, we examined how four previously-determined key factors to winning^14,15^ differed when a team traveled from their home location to locations within and across time zones (i.e., the travel effect) as well as between travel within the team’s home time zone to travel to other time zones (thereby standardizing for travel, opposing crowds, etc. [i.e., the time zone effect]). Specifically, we examined the shooting metric effective field goal percentage (eFG%; adjusts for 3-point field goals being worth more than two-point field goals; Fig. 2a), number of free-throws attempted per field-goals attempted (free-throw rate; Fig. 2b), the percentage of offensive rebounds a team obtains (ORB%; Fig. 2c), and the percentage of possessions a team turns-over the basketball to the opposing team per 100 possessions (turnover%; Fig. 2d). We also examined overall offensive (i.e., points scored per 100 possessions; Fig. 2e) and defensive ratings (i.e., points allowed per 100 possessions; Fig. 2f). As compared to when playing at home, we found decreases in eFG% at two time zones (− 1.5%, t(21) = 2.8, p = 0.01; Fig. 2a), turnover% at two (− 0.83%, t(21) = 2.9, p < 0.01) and three (− 1.6%, t(21) = 2.8, p = 0.02) time zones (Fig. 2d), and ORB% at all time zones (all p < 0.026; Fig. 2c). Additionally, we found that defensive rating was significantly higher (i.e., worse performance) at all time zones (≥ 2.8 more points allowed per 100 possessions; all p < 0.021; Fig. 2f). When we compared travel from within the team’s time zone to traveling to other time zones, we found that eFG% of teams traveling two time zones was significantly decreased by 1.6% (t(11) = 2.6, p = 0.02; Fig. 2a), and turnover% decreased by 1.5% (t(11) = 2.6, p = 0.02) when traveling three time zones (Fig. 2d).

Lastly, to understand the impact of home-court advantage, we compared in-game performance metrics between playing at home pre-COVID-19 to playing within the COVID-19 regular season bubble (combining the “home” and “away” designations). We found that free-throw attempt rate (+ 2.6%, (t(21) = 3.2, p < 0.01; Fig. 2b) and defensive rating (~ 5.5 more points allowed per 100 possessions, (t(21) = 5.0, p<0.0001; Fig. 2f) both significantly increased in the bubble setting as compared to their home arena pre-COVID-19.

## Discussion

The advantages of playing a sporting event at a home venue have long been recognized, but the ability to uncover the impact of the opposing team traveling (often multiple time zones) vs the benefits of playing at home are not clear. Using the natural experiment provided by the NBA COVID-19 shut-down and subsequent bubble restart, we could systematically examine which aspects of regular season team performance changed when teams were traveling within and across time zones, and when no teams traveled (bubble). We found that winning percentages only significantly differed when traveling across time zones, predominately associated with teams traveling westward, and this was accompanied by decreases in teams’ shooting accuracy (i.e., eFG%), effort (ORB%), turnover%, and increases in points allowed (defensive rating). Importantly, eFG% and turnover% also differed when comparing travel without changing time zones with travel changing time zones, suggesting there is a specific time zone/circadian effect on these metrics in addition to a general travel effect. Lastly, when examining performance in the bubble, we did not observe a significant difference in winning percentage dependent on teams’ home vs away designation, though there was a non-significant trend for the designated away team to win a lower percentage of games. Thus, circadian effects and travel cannot fully account for home and away differences. Moreover, we found that out of the four key factors to winning^14^, only free-throw rate increased as compared to home games pre-COVID-19. Taken together, the combination of travel and circadian disruption during regular season gameplay might impair the away team’s ability to rebound and shoot accurately, which may partly account for home-court advantage in professional basketball. These data may also suggest that different aspects of basketball performance are influenced differently by travel and circadian disruption, which may be important knowledge when developing future strategies for playing at an opponent’s arena and creating travel schedules that could better cater to performance (i.e., timing of events for circadian adaptation).

By comparing metrics across time zones relative to travel within a time zone, and not just compared to the home arena, we were able to control for the general influence of travel and thereby examine potential circadian influences. Similar to previous reports, we observed that winning was impacted by westward travel^16,17^, however we can now hypothesize that this may be in part due to reductions in shooting accuracy by the away team. Moreover, examinations of home-advantage have identified increased points scored^18^ at home versus away, which is related to shooting accuracy and is in accordance with our findings of increased defensive rating as compared to home. Decrements in accuracy have also been reported in other types of elite athletes, namely competitive marksmanship, when traveling multiple time zones^19^. In professional baseball, the greater number of time zones a team travels has been found to be associated with winning percentage^17^, potentially driven by impaired pitching accuracy as observed through an increased number of home runs allowed by a jet-lagged pitcher^3^. Potential reasons for decreased accuracy when traveling could be from the sleep disturbance associated with discomforts while traveling and the circadian disruption of changing time zones (i.e., jetlag)^20^, as extending sleep has been shown to improve shooting accuracy^21^. Other explanations could include players performing at circadian times that are not optimal for performance, as it has been shown that athletic performance is strongly driven by circadian timing, particularly in elite athletes exerting maximal effort^2^. The fact that we did not find shooting accuracy to decrease in the bubble, yet defensive rating did, may suggest that because no team was traveling (and thus shooting more poorly as the traveling team), more points could be scored by the opposing team. These findings of decreases in accuracy when traveling across multiple time zones may be applicable to other professional sports in which accuracy is paramount. As mentioned above, reductions in accuracy from travel could have implications for elite baseball players and account for changes in winning when playing at home or away^3^. The sports league with the most similarities to the NBA schedule is the National Hockey League (NHL), which also consists of 82 regular-season games and an approximate 90% overlap in the actual months in which they play (October to June). The NHL also has other similarities to the NBA such as the indoor arena environment (in fact, many of the NBA teams actually share the same arena as the city’s NHL team) and that teams also display a circadian disadvantage when traveling westward^16,22^. Thus, our findings may provide information that could be beneficial to teams beyond professional basketball when developing strategies for performing at the highest level when traveling. Moreover, our findings from this unique natural experiment may provide specific examples of the consequences of travel and jetlag in basketball and could be measured for improvement after implementation of suggested interventions^5,7,23,24^ to combat jetlag for peak performance.

Despite utilizing a natural experiment, there are several limitations to consider within the analysis. We only had data available from the relatively small sample size of regular season games played in the bubble. Relatedly, these were the 22 most winning teams pre-COVID-19, and it is likely that they had better performance parity than if all 30 teams were included. Additionally, though we did not include playoff games, team playing strategies may have still differed within the restart bubble due to factors such as environmental differences (e.g., smaller arena, no live crowd), player health/injury status, and incentive to win (e.g., some teams had already clinched their playoff position), among other possible differences. These playing strategies may have also differed due to members of certain teams opting-out of partaking in the bubble, COVID-19 related illness impacting players’ performance, or other bubble-related reasons that could not be accounted for in the current analysis. In the pre-COVID-19 games, strong effects were not found for teams traveling three time zones, even though circadian disruption and travel are greatest under that condition. Because only a subset of teams (based in Eastern or Pacific time zones) can travel three time zones *and* can only do so in one direction, the results for 3 time zones and directional travel were limited by these sample size restrictions. We chose not to include any previous season’s data to our analysis for increased sample size and power as we would be unable to directly compare variables in the same team’s performance from during the pre-COVID-19 regular season with the changes that occurred within the restart bubble. Moreover, we were unable to identify the exact travel schedule of each team, so it is unknown how long a team had been in any particular time zone or how rapidly they had changed time zones before playing in their respective games. Additionally, different teams may use different strategies to combat the deleterious impacts of travel (e.g., travel to the away location the night before a game vs. the day of the game or institute pre-game rest intervals). Although there were no data available on the actual sleep patterns of teams or players at any point in the season, strategies to combat the effects of travel may have included changes to the duration and timing of sleep itself. Sleep in the bubble could have been changed (either positively or negatively) from sleep patterns in the pre-COVID-19 regular season, depending on how teams and players responded to the novel living conditions and altered schedule of practices and games in the bubble environment. Thus, the degree of predicted circadian (and sleep) disruption for teams may have been attenuated in some instances. We also did not have data at the individual level regarding each player’s internal circadian timing, and therefore individual differences in circadian disruption could have differed between players and affected team outcomes. Lastly, we also cannot rule out the possibility that differences within the bubble may have also been under-powered to detect, had each team been scheduled to play more than just eight regular season bubble games. Despite these limitations, our findings from the analyses of restart bubble games are consistent with hypothesized performance changes (e.g., reducing home-court winning advantage) due to removing the travel/circadian factors, and therefore our findings lend support to the motivation for conducting the study of this natural experiment.

In conclusion, our findings using a natural experiment demonstrate that athletic competition in a scenario where no teams travel reduces the traditionally observed home-court advantage on winning percentage, shooting accuracy, and rebounding in professional basketball. Importantly, these advantages appear to be linked with the away team’s impaired shooting accuracy when traveling multiple time zones and rebounding when traveling in general. Future work is needed to explore other athletic events where travel schedules can impact performance to further elucidate travel and circadian factors.

## Materials and methods

We retrospectively examined each of the 22 NBA teams invited to the regular season restart bubble and compared their winning and athletic performance among three playing scenarios: (1) games played at home before the COVID-19 shutdown (649 games total), (2) games played when traveling “away” between 0 and 3 time zones before the COVID-19 shutdown (715 games total), and (3) games played after all teams lived and played in the same location (bubble) for their final eight regular season games (176 games total). For our primary analysis, we chose to focus on only the subset of 22 restart teams, instead of including all 30 NBA teams, in order to directly compare their pre-COVID-19 team performance to then playing within the bubble. Moreover, we did not include any of the playoff games that occurred within the restart bubble so as to not confound the findings with any potential changes made arising from either playing with more effort and/or caution during higher-stakes playoff setting (e.g., “trying” harder to play at peak performance, being more careful with possession of the ball, etc.) or playing the same team multiple times in a row (e.g., playing strategy) which could potentially mitigate travel impairments.

Game locations, team results, and statistics were obtained from the freely-available www.Basketball-Reference.com^25^, a website where data are published by the official statistics provider of the NBA (Sportradar; St. Gallen, Switzerland). The average age of the 22 teams that partook in the bubble was 25.6 years (± 0.33 standard error of the mean, range 23.5–29.0 years). Only games that counted towards a team’s final record were used. Each game in the pre-COVID-19 regular season and during the COVID-19 restart bubble were examined individually for each team’s performance and time zone changes were matched based on the home-team’s geographical location. Daylight saving time was factored-in for time zone for games played in Phoenix, Arizona, which remains in standard time year-round, such that games played in the first two weeks of the season (from the October start until November 3rd) were binned into the Pacific time zone and games played thereafter were binned into the Mountain time zone. Pre-COVID-19 games played in Mexico (n = 1) and France (n = 1) were not included in analysis due to other potential factors associated with international travel affecting both teams and not having a true home or away team venue.

Advanced basketball statistics (e.g., the four factors of winning) were chosen due to their previous association with winning percentage and to standardize games and/or teams that play at a different pace^14,15^. In brief, EFG% weights shooting percentage to account for the fact that a three-point field goal is worth more than a two-point field goal (field goal made + 0.5 × three-point field goals made)/field goals attempted). Turnover% is an estimate of the number of turnovers a team makes over 100 possessions, thus standardizing for pace of play (100 × turnovers/(field goal attempts + 0.44 × free-throw attempt + turnovers). ORB% is the percentage of rebounds a team obtains from any field-goals and free-throws attempted by their own team (100 × (offensive rebounds × (minutes the team has played/5))/(minutes played × (team offensive rebounds + opponents’ defensive rebounds)). Lastly, free-throw attempt rate is the number of free-throws attempted per the number of field goals attempted (free-throw attempts/field-goal attempts)^25^.

Winning percentages were analyzed using linear mixed effects models with location as a fixed factor and team as a random factor. After testing all data for normality using skewness and kurtosis metrics, planned comparisons were conducted between locations for winning percentages and advanced in-game playing statistics using two-tailed dependent t-tests with modified Bonferroni correction for multiple planned comparisons to reduce type 1 error^26^. Pearson correlation coefficient was used to determine the association between the total number of time zones traveled for games pre-COVID-19 and winning percentage.

## Data Availability

All data are freely available on www.basketball-reference.com^25^.
